# Nursing Support for Exercise Adherence in Chronic Disease Management: A Qualitative Descriptive Study of Patient and Nurse Perceptions

**DOI:** 10.3390/healthcare14182980

**Published:** 2026-09-12

**Authors:** Jamal M. Alzahrani, Abdulaziz M. Alodhailah, Abdullah Alharbi, Bandar S. Alharbi, Monirah Albloushi, Mohammed Almutairi

**Affiliations:** 1 Exercise Physiology Department, College of Sport Sciences and Physical Activity, King Saud University, Riyadh 11451, Saudi Arabia; 2 Department of Medical-Surgical Nursing, College of Nursing, King Saud University, Riyadh 11451, Saudi Arabia; 3 Department of Community and Psychiatric Mental Health Nursing, College of Nursing, King Saud University, Riyadh 11451, Saudi Arabia

**Keywords:** exercise adherence, physical activity, chronic disease management, nursing support, qualitative descriptive study, reflexive thematic analysis, patient experience, Saudi Arabia

## Abstract

**Background**: Physical inactivity is common among people living with chronic non-communicable disease, and nurses are frequently the professionals with the most sustained patient contact. How nursing support for physical activity is experienced by patients, and how nurses understand their own role in providing it, remain poorly described. **Objective**: To explore how patients with chronic diseases and nursing professionals describe nursing support for physical activity and exercise adherence, and where their accounts converge or diverge. **Methods**: A qualitative descriptive study using individual semi-structured interviews and reflexive thematic analysis. Seventeen participants (nine patients with chronic diseases and eight nursing professionals) were purposively recruited from five healthcare institutions in the Riyadh region of Saudi Arabia. Interviews lasted 30 to 60 min (mean 45 min) and were conducted between December 2025 and February 2026. No quantitative measures of exercise frequency, intensity, duration, or session regularity were collected, so the study reports perceptions and experiences rather than measured adherence, and no causal relationships were examined. Rigor was addressed through researcher reflexivity, member checking, peer debriefing, and an audit trail; reporting follows COREQ. **Results**: Five themes were constructed: (1) Nursing-Facilitated Empowerment Through Education and Guidance; (2) Personalized Support and Accountability Mechanisms; (3) Barriers and Facilitators to Exercise Adherence; (4) Perceived Health Benefits and Motivation Enhancement; and (5) Gaps in Current Nursing Practice and Opportunities for Enhancement. Participants identified individualized explanation, graduated goal-setting, correction of fear-based misconceptions, scheduled exercise-specific review, and family involvement as the components of support they valued most, and lapsed follow-up, generic advice, limited interdisciplinary coordination, and scarce patient resources as the principal shortfalls. **Conclusions**: Patients and nurses in this setting described sustained, individualized nursing support as central to how patients engage with exercise, while both groups identified structural constraints that limit it. These accounts indicate targets that intervention studies with objective activity measurement could test; they do not establish that nursing support changes exercise behavior.

## 1. Introduction

Chronic non-communicable diseases are the leading cause of death worldwide. They killed at least 43 million people in 2021, approximately three quarters of all non-pandemic-related deaths, and 18 million of those deaths occurred before the age of 70 [1]. Progress towards the Sustainable Development Goal target of reducing premature mortality from these conditions remains off track in most countries, including those of the Middle East and North Africa [2]. Within Saudi Arabia, a national survey conducted in 2020 estimated the weighted prevalence of obesity (body mass index ≥ 30 kg/m^2^) at 24.7%, with obesity significantly associated with type 2 diabetes, hypertension, and hypercholesterolemia [3].

Physical activity and structured exercise are cornerstone components of chronic disease management, although the two terms denote different behaviors and are frequently used interchangeably in the clinical literature [4]. Qualitative work with older adults living with type 2 diabetes indicates that whether people act on activity advice depends on disease-related, psychological, and social influences rather than on knowledge alone [5]. Nurse-led approaches that individualise this support have improved treatment adherence and self-management in chronic disease populations [6].

Adherence to recommended activity levels nonetheless remains low, and the distance between understanding the value of physical activity and sustaining it is well documented [7]. Reviews of barriers and motivators among middle-aged and older adults consistently identify fear of injury or symptom exacerbation, pain, low motivation, competing time demands, limited social support, and inaccessible or costly facilities as the dominant determinants of participation [7,8].

Nurses, as frontline healthcare providers with extended patient contact and established therapeutic relationships, are well placed to address this adherence gap. Even within structured, professionally supervised programs such as cardiac rehabilitation, attendance and continued participation are shaped by psychosocial and practical factors as much as by clinical need [9]. Nurses are well placed to deliver holistic, patient-centered interventions that address both the biological and psychosocial dimensions of exercise adherence. However, substantial variation exists in how nurses approach physical activity promotion, and limited empirical evidence describes the mechanisms through which nursing support is experienced as helpful by patients themselves. Interventions that raise health literacy improve health literacy, self-efficacy, and self-reported health status, but their effects on disease-specific self-care behaviors are inconsistent and often attenuate within three months [10].

Furthermore, while individual patient and nurse perspectives have been explored separately in existing literature, few studies have examined both viewpoints simultaneously within a single research context. A dual-perspective approach gives a fuller account of the nurse–patient dynamic around exercise promotion, showing where intended nursing interventions and patient experience align and where they do not. This approach is consistent with person-centered care paradigms that emphasize collaborative understanding and shared decision-making.

### 1.1. The Burden of Chronic Diseases and the Role of Exercise

Chronic diseases represent the predominant health challenge of our era. The burden extends beyond individual health outcomes to the capacity of health systems to respond. Surveillance and prevention infrastructure for these conditions remains uneven across high-burden countries, which constrains both monitoring and response [11]. In Saudi Arabia, national health statistics document the scale of chronic disease service provision within the public health system and the volume of outpatient contacts associated with it [12].

Physical activity serves as a cornerstone intervention across all major chronic disease management guidelines. The World Health Organization, American Heart Association, American Diabetes Association, and numerous national health bodies consistently recommend at least 150 min of moderate-intensity aerobic activity weekly for adults, including adults living with chronic conditions or disability [13]. Current guidance also holds that some physical activity is better than none, so that even modest increases, when sustained, are expected to confer benefit [13].

However, the translation of guideline recommendations into sustained patient behavior remains incomplete. Reported adherence estimates vary widely across studies, in part because adherence is defined and measured inconsistently, with some studies counting attendance at supervised sessions and others self-reported weekly minutes [4,7]. Sustained non-adherence is associated with suboptimal disease control, preventable complications, increased healthcare utilisation, and reduced quality of life.

### 1.2. Conceptual Understanding of Exercise Adherence

Exercise adherence, defined as the degree to which individuals follow a recommended physical activity regimen, is a multifactorial phenomenon that linear biomedical models explain poorly. Three complementary frameworks informed this study. Social Cognitive Theory holds that behavior, personal factors (notably self-efficacy beliefs and outcome expectations), and environmental factors influence one another reciprocally, so that a person’s confidence in their ability to exercise despite chronic disease constraints becomes a primary determinant of whether they begin and continue [14]. Self-Determination Theory proposes that sustained behavior change depends on the satisfaction of three psychological needs, competence, autonomy, and relatedness; where these are met within the clinical encounter, patients are more likely to move from externally motivated compliance towards self-endorsed engagement [15]. Pender’s Health Promotion Model positions behavior-specific cognitions, including perceived benefits, perceived barriers, and interpersonal influences, as the most proximal determinants of health-promoting action such as exercise [16]. These three frameworks were used to orient the interview guide and to inform interpretation of the constructed themes. They were not used to pre-code the data, and the study does not test any proposition derived from them.

Terminology in this field is applied inconsistently, and fixed definitions are therefore adopted here. Physical activity refers to any bodily movement produced by skeletal muscle that expends energy. Exercise refers to the planned, structured, repetitive subset of physical activity undertaken to improve or maintain fitness. Exercise adherence refers to the degree to which a person sustains a recommended activity regimen over time [4]. The term exercise compliance is retained only where cited authors used it themselves, and is discussed in the following paragraph. These definitions are applied consistently throughout the manuscript.

A critical distinction exists between exercise compliance, a term implying passive adherence to externally imposed prescriptions, and exercise adherence, which reflects active, voluntary engagement in exercise aligned with personal goals and values. Contemporary healthcare approaches emphasize adherence, recognizing that sustainable behavior change emerges when individuals internalize the importance of behavior change and perceive autonomy in implementing it [15].

### 1.3. Nursing’s Contribution to Health Behavior Change

The nursing profession, grounded in humanistic care principles and equipped with knowledge spanning biological, psychological, and social domains, is well placed to support health behavior change. The therapeutic nurse–patient relationship, characterized by trust, empathy, and collaborative problem-solving, provides the foundation for this work [17]. Nursing interventions for behavior change typically combine health education, motivational dialogue, collaborative goal-setting, barrier identification, monitoring and feedback, and psychosocial support [10,18].

A nurse-led program built explicitly on Pender’s Health Promotion Model improved exercise behavior among patients with coronary artery disease, with self-efficacy identified as the mechanism through which the program operated [18]. Proposed mechanisms for nurse-led adherence support more generally include improved health literacy, increased self-efficacy, strengthened motivation, facilitated social support, and structured accountability. Substantial heterogeneity nonetheless exists in how nurses approach physical activity promotion: some emphasize didactic health education, others motivational support, and others barrier identification and problem-solving. The extent to which these varied approaches align with patient needs and preferences remains inadequately explored.

### 1.4. Gaps in Existing Literature

Current literature on exercise adherence in chronic disease populations predominantly comprises quantitative studies examining the efficacy of specific interventions or the statistical relationships between variables and adherence outcomes. While valuable, this literature rarely captures how adherence unfolds in real-world contexts, how patients experience nursing support, or which mechanisms patients themselves perceive as most influential.

Qualitative research addressing exercise adherence in chronic disease populations exists but remains relatively scarce. Existing qualitative studies have predominantly focused on either patient perspectives (examining barriers, facilitators, and experiences with exercise) or nurse perspectives (exploring how nurses conceptualize their role in health promotion), but rarely have both perspectives been examined within a single study context. Additionally, much existing qualitative research on exercise adherence has been conducted in high-income English-speaking countries, limiting its applicability to diverse cultural and healthcare contexts.

Furthermore, the intersection of nursing support and patient experience in exercise adherence, specifically how nursing interventions are perceived by patients and how nurses understand their own effectiveness, remains under-researched. Understanding this dyadic process is essential for developing nurse-led interventions that are not only theoretically sound and evidence-based but also aligned with patient needs, preferences, and lived experiences.

### 1.5. Aim of the Study

This study aimed to explore patients’ experiences with physical activity and the nursing support they received in the context of chronic disease management, alongside nurses’ perspectives on their role in promoting and maintaining exercise adherence. Specific research questions guiding this inquiry were:

(1) What are the lived experiences of patients with chronic diseases regarding exercise participation, and how do they perceive the contribution of nursing support to their physical activity engagement?
(2) What individual, social, and environmental barriers and facilitators shape exercise adherence among patients with chronic diseases, and how do nursing interventions address or fail to address these factors?
(3) How do nursing professionals conceptualize their role in facilitating exercise adherence, and what specific strategies, challenges, and resources shape their approach to physical activity promotion?
(4) In what ways do patient experiences of nursing support for exercise adherence converge with or diverge from nurses’ perceptions of their own practice, and what are the implications of these convergences and divergences for person-centered care?

## 2. Materials and Methods

### 2.1. Research Design

This study used a qualitative descriptive design with reflexive thematic analysis as the analytic approach. Qualitative description was selected because it allows phenomena to be described as experienced and understood by participants without imposing a predetermined theoretical framework [19,20]. The design is interpretive and is not a cross-sectional survey: no variables were measured, no hypotheses were tested, no statistical inference was undertaken, and no causal relationship is examined or claimed anywhere in this report. Reporting follows the Consolidated Criteria for Reporting Qualitative Research (COREQ), and the completed 32-item checklist is provided as Supplementary Table S1 [21].

Reflexive thematic analysis was selected as the analytical approach. This approach explicitly acknowledges the researcher’s active role in constructing meaning from data and emphasizes the iterative, interpretive process through which themes are identified and developed. Reflexive thematic analysis provides structured yet flexible procedures for developing rich, contextually grounded understandings of phenomena while maintaining rigor through systematic data engagement, transparent decision-making, and explicit attention to researcher positioning [19].

### 2.2. Setting and Participants

#### 2.2.1. Setting

The study included participants from different healthcare institutions in the Riyadh region of Saudi Arabia (three government tertiary hospitals and two private specialty clinics), encompassing both government and private facilities. The institutions’ chronic disease management services include outpatient clinics for diabetes, hypertension, cardiovascular disease, and obesity, staffed by nursing professionals with varying levels of education and experience in chronic disease management.

#### 2.2.2. Participant Selection and Sampling Strategy

A purposive sampling strategy was employed to recruit participants with rich information regarding the phenomenon of interest: nursing support for exercise adherence in chronic disease management. Sampling continued until informational redundancy was achieved, wherein successive interviews ceased to yield new codes or thematic categories; this point was reached after 15 interviews, with the final two participants confirming rather than extending the established thematic structure. Given the narrow and clearly specified aim, the use of established theory to structure the interview guide (Table S2), the dual-participant composition, and the quality of the interview dialogue achieved, the information power of a sample of 17 was judged adequate for this aim [22]. The sample was not designed to support statistical inference, and its size is revisited in Section 4.9. Participants comprised two groups:

##### Group 1: Patients with Chronic Diseases (*n* = 9)

A patient group of nine was determined through purposive maximum-variation sampling targeting diversity across chronic disease type, disease duration, physical activity level, age, sex, and educational background. Sampling for this group targeted richness of perspective rather than statistical representativeness, consistent with information power reasoning [22]. Recruitment ceased when successive interviews confirmed no new codes or thematic categories were emerging. Inclusion criteria were: (1) diagnosis of at least one chronic disease (including type 2 diabetes mellitus, hypertension, cardiovascular disease, obesity, or combinations thereof); (2) age ≥18 years; (3) capacity for light-to-moderate physical activity as assessed by their healthcare provider; (4) current receipt of care from the study sites’ chronic disease management services; and (5) fluency in Arabic or English. Exclusion criteria included: (1) acute illness or hospitalization; (2) cognitive impairment precluding informed consent; (3) terminal illness; and (4) unwillingness to provide informed consent.

##### Group 2: Nursing Professionals (*n* = 8)

Inclusion criteria were: (1) registered nurse or nurse specialist providing care to patients with chronic diseases; (2) minimum of one year of experience in chronic disease management; (3) current employment at one of the study sites; and (4) fluency in Arabic or English. Exclusion criteria included: (1) administrative nursing roles without direct patient care responsibilities; and (2) unwillingness to provide informed consent.

Participants were recruited through chronic disease outpatient clinics using flyers, information sheets, and verbal announcements, supplemented by social media and professional networking channels. Snowball sampling was also employed. Interested patients and nurses were screened for eligibility and provided with detailed study information, and written informed consent was obtained prior to interview scheduling.

Purposive sampling in qualitative research is not designed to produce a statistically representative sample; rather, it aims to generate a sample rich in perspectives relevant to the research phenomenon. The combination of purposive and snowball sampling in this study was deliberate: purposive selection ensured diversity across key participant characteristics, while snowball sampling reached nursing professionals and patients who might not respond to institutional recruitment alone. These strategies are complementary and commonly employed together in qualitative nursing research [19,20]. The non-representativeness of the sample is an inherent and appropriate feature of the descriptive qualitative design; findings are not intended to be generalized statistically but to generate conceptual insights applicable to similar chronic disease nursing contexts.

### 2.3. Data Collection

Semi-structured interviews were conducted from December 2025 through February 2026. Interviews were selected as the primary data collection method as they allow deep exploration of personal experiences, beliefs, motivations, and perspectives within a conversational context that facilitates disclosure and meaning-making [20].

Data collection was confined to participants’ verbal accounts. No objective or self-reported quantitative measure of exercise frequency, intensity, duration, session regularity, or energy expenditure was collected. No activity diary, pedometry, accelerometry, or exercise-testing data were obtained, and no clinical or biochemical outcome data were extracted from medical records. Where participants referred to their own activity, for example, walking four times each week, these were self-reported statements made during the interview and were not verified against any independent record. The study therefore reports how participants described exercise adherence and the nursing support surrounding it, and does not measure adherence itself. This boundary is carried through the Results, Discussion, and Conclusions, and its consequences for interpretation are set out in Section 4.9.

A semi-structured interview guide with open-ended primary questions and flexible probing questions was developed by the research team, informed by existing literature on exercise adherence, health behavior change, and nursing interventions. The interview guide for patient participants included 20 primary questions addressing: (1) overall experiences with exercise; (2) experiences with nursing support; (3) types of information and guidance provided by nurses; (4) facilitators and barriers to exercise adherence; (5) effects of nursing support on motivation; (6) perceived monitoring and accountability; (7) encounter difficulties and nursing responses; (8) perceived health benefits; (9) appropriateness of recommendations; (10) desired changes in nursing support; (11) effects of exercise on daily life; (12) future expectations from nursing; (13) whether nurses understood their needs; (14) most valued aspects of nursing support; (15) areas for improvement; (16) willingness to recommend exercise to others; and (17) recommendations for nursing programs.

The interview guide for nurse participants addressed parallel themes adapted for the nursing perspective, including: (1) conceptualization of their role in promoting exercise; (2) strategies used to support patients; (3) perceived barriers to exercise adherence in their patient populations; (4) approaches to tailoring interventions to individual patients; (5) challenges encountered in promoting exercise; (6) perceived effectiveness of their interventions; (7) desired professional development or resources; and (8) recommendations for improving nursing support for exercise adherence. Representative verbatim items from the patient guide included: “Can you describe what exercise or physical activity looks like in your daily life at the moment?”; “Can you tell me about a time when a nurse or healthcare provider helped you with your exercise routine: what did they do and how did it affect you?”; and “What are the main things that make it difficult for you to exercise regularly, and how have nurses helped you deal with these?” Representative verbatim items from the nurse guide included: “How would you describe your role in supporting patients with chronic diseases to be physically active?”; “What specific strategies do you use to encourage patients to start or maintain an exercise routine, and how do you adapt these to different patients?”; and “What do you see as the biggest barriers to providing effective exercise support in your clinical setting?” The complete interview guides are available from the corresponding author on reasonable request.

Interviews were conducted by trained research team members with prior qualitative research experience, either in a quiet, private location chosen by each participant or via secure online platforms. Interviews were led by A.M.A. (PhD), who had no prior relationship with any participant before the study. No one other than the participant and the interviewer was present during interviews, and no repeat interviews were conducted. Interviews lasted between 30 and 60 min, with a mean duration of 45 min across the whole sample. Interviews with patients and with nurses were scheduled on the same basis and drew on parallel guides of comparable length. All interviews were audio-recorded with participant consent and transcribed verbatim in their original language (Arabic). Arabic transcripts were then translated into English by a bilingual research team member, with accuracy verified through independent back-translation procedures: a second bilingual research team member, blind to the original Arabic transcripts, independently translated the English versions back into Arabic. The two Arabic versions (original transcripts and back-translated text) were then compared by a third bilingual team member to identify any semantic discrepancies. Minor lexical differences were resolved through consensus discussion among the three team members; no substantive meaning discrepancies were identified.

### 2.4. Researcher Reflexivity and Positioning

Reflexivity, the process through which researchers explicitly acknowledge, examine, and account for their subjectivity, values, and potential influence on research processes and findings, constitutes a cornerstone of qualitative research rigor [19]. The research team engaged in multiple reflexive practices throughout this study.

The lead researcher, a nurse with 15 years of clinical nursing experience and 8 years of research experience, brought substantial knowledge of nursing practice, chronic disease management, and health behavior change. This background provided valuable context for understanding nurse and patient perspectives but required deliberate bracketing of assumptions about “ideal” nursing practice to maintain openness to diverse participant perspectives. Throughout data collection and analysis, the lead researcher kept a reflexive journal documenting emerging insights, assumptions, and interpretations.

The research team held regular debriefing meetings during data collection and analysis to discuss emerging themes, identify potential researcher biases, and ensure diverse interpretations of data were considered. The team explicitly discussed their professional backgrounds, personal experiences with exercise, and beliefs about nursing’s role in health promotion to heighten awareness of how these factors might influence data interpretation.

Data analysis was undertaken collaboratively, with initial coding conducted independently by multiple team members who then compared and discussed coding decisions. This collaborative approach ensured that interpretations were not unduly influenced by any single researcher’s perspective and that alternative interpretations were actively considered.

### 2.5. Data Analysis

Data analysis employed reflexive thematic analysis as operationalized by Braun and Clarke (2019) [19], an approach that conceptualizes themes as patterns of shared meaning across data associated with a particular research question. Reflexive thematic analysis proceeds through six iterative phases:

Phase 1: Familiarization with Data. Researchers individually read and reread each transcript, listening to audio recordings while following along with written transcripts to enhance familiarity with data nuances. During this immersion process, researchers noted initial observations, striking phrases, patterns, and areas of interest.

Phase 2: Initial Coding. Using NVivo 14 qualitative analysis software, researchers systematically coded all data. Coding was data-driven rather than predetermined, with codes generated from explicit content of participant responses. For example, when a patient described how a nurse helped them set realistic exercise goals, this segment was coded “Goal-setting support”. When a nurse discussed barriers patients face, codes captured specific barriers mentioned (e.g., “Fear of symptom exacerbation”, “Lack of motivation”). Both semantic codes (capturing surface-level meanings) and latent codes (capturing underlying assumptions and ideologies) were generated. NVivo 14 was used specifically for data management and organizational purposes. All 17 interview transcripts were imported into a single NVivo project. Each researcher independently created coding nodes within the project, applied codes to transcript segments, and recorded memos documenting their coding rationale. The project’s shared file structure allowed all team members to view and compare one another’s coding decisions before consensus meetings. Coded nodes were then exported and reviewed collectively to identify patterns, redundancies, and candidate themes. It is emphasized that NVivo served as a tool for organising and retrieving coded data; all interpretive and thematic decisions were made by the research team through iterative discussion rather than by software.

Phase 3: Searching for Themes. Coded data segments were grouped and organized into broader thematic categories capturing patterns of meaning. During this phase, researchers examined relationships between codes, identified hierarchical structures (overarching themes encompassing multiple subthemes), and generated candidate themes.

Phase 4: Theme Refinement and Development. Candidate themes were refined through multiple rounds of iterative analysis. For each theme, researchers reviewed the full dataset to identify all relevant data segments, assess internal consistency (coherence among codes within a theme), and ensure adequate distinction from related themes. Themes that lacked sufficient supporting data collapsed into broader themes, and themes lacking internal coherence were separated into distinct themes.

Phase 5: Theme Naming and Definition. Each finalized theme was assigned a concise, descriptive name capturing its essence. Detailed theme definitions were developed, articulating the boundaries of each theme, its key characteristics, and how it related to other themes.

Phase 6: Theme Integration and Reporting. Themes were organized conceptually to reflect their relationships and significance to the research questions. Themes are presented in the Findings section with rich illustration through participant quotes and analytic narrative.

Throughout the analysis, systematic attention was paid to both convergences and divergences between patient and nurse perspectives. Particular attention was given to areas where patient experiences and nurse perceptions aligned, as well as areas of disconnection that might inform recommendations for practice improvement.

Transcript length and depth varied between participants. All 17 transcripts were coded and every transcript contributed codes to the final thematic structure; none was excluded on grounds of insufficient depth. Richness was not formally rated, however, and the more extended accounts inevitably supplied a greater share of the illustrative extracts presented in Section 3.3.

### 2.6. Trustworthiness and Rigor

Multiple established strategies were employed to enhance credibility, dependability, and confirmability of findings:

Credibility was enhanced through (1) prolonged engagement with the data; (2) persistent observation, involving extensive time spent in the interview contexts and during data analysis; (3) member checking, whereby preliminary findings were shared with a purposively selected subset of participants (*n* = 4 patients, *n* = 3 nurses) to assess interpretive accuracy; (4) peer debriefing, through regular meetings with the research team to consider alternative interpretations and critically examine emerging findings; and (5) participant review of a subset of transcripts, whereby two transcripts were returned to participants for review and comment on their accuracy before analysis. This transcript review was distinct from the subsequent member checking of preliminary findings.

Dependability was ensured through (1) detailed documentation of all methodological decisions and their rationale; (2) transparent description of data collection and analysis procedures; and (3) audit trail maintenance, with all raw data, analytical decisions, and thematic development systematically documented for potential audit.

Confirmability was enhanced through (1) reflexive bracketing of researcher biases and assumptions; (2) collaborative analysis involving multiple researchers; and (3) explicit documentation of how conclusions emerged from data rather than from researcher preconceptions.

Transferability was addressed through provision of rich, detailed descriptions of participant characteristics, research context, and findings, enabling readers to assess applicability to other populations and settings.

### 2.7. Ethical Considerations

Ethical approval was obtained from the King Saud University Institutional Review Board (KSU-HE-25-1489; approved 16 December 2025). Written informed consent was obtained from all participants, and participation was voluntary.

All participants provided written informed consent following a detailed explanation of study purposes, procedures, risks, benefits, and rights. Particular attention was paid to ensuring that patients understood that study participation was entirely voluntary and that declining participation would not affect their clinical care. Nursing participants were assured that their participation would not influence employment or professional evaluations.

Confidentiality was rigorously protected through multiple safeguards: (1) all identifiable participant information was removed from transcripts and replaced with participant identification numbers; (2) all electronic data were stored on password-protected computers accessible only to authorized research team members; (3) audio recordings were stored separately from transcripts and identifying information; and (4) any direct quotes used in reporting were carefully reviewed to ensure they contained no identifiable information. Stored data will be retained for five years following study completion by institutional requirements and then securely destroyed.

Participants were informed of their right to withdraw from the study at any time without consequences. Two individuals approached during recruitment indicated that they preferred not to participate; their decision was respected and no follow-up recruitment was attempted. Consistent with the approved consent procedure, no demographic, clinical, or experiential information was collected from those who declined. It is therefore not possible to establish whether their experiences of nursing support differed from those of the participants, and this constraint is addressed in Section 4.9.

## 3. Results

### 3.1. Participant Characteristics

A total of 17 participants were enrolled and completed interviews. The patient group (*n* = 9) ranged in age from 38 to 72 years (M = 54.2, SD = 11.8), with five females and four males. Six participants reported type 2 diabetes mellitus, five reported hypertension, one reported cardiovascular disease, and three reported multiple chronic conditions (diabetes and hypertension, or hypertension and cardiovascular disease). Disease categories are not mutually exclusive, so these counts sum to more than nine. No participant reported chronic obstructive pulmonary disease, malignancy, or inflammatory arthritis. Disease duration ranged from 2 to 18 years. Regarding current physical activity levels, three patients reported low activity levels, four reported moderate activity levels, and two reported higher activity levels. Educational backgrounds ranged from primary education to university-level education. Four participants were employed, and five were retired or unemployed.

The nursing group (*n* = 8) ranged in age from 29 to 58 years (M = 42.1, SD = 10.4), with six females and two males. Educational qualifications ranged from nursing diploma to master’s degree: three participants (37.5%) held a nursing diploma, two (25.0%) held a bachelor’s degree in nursing, and three (37.5%) held a master’s degree in nursing. All participants held a minimum qualification of registered nurse recognized by the Saudi Commission for Health Specialties. Years of experience in nursing ranged from 4 to 28 years (M = 13.8, SD = 8.2), with all participants having at least 2 years of experience in chronic disease management. Five nurses worked in outpatient chronic disease clinics, and three worked in inpatient units caring for patients with chronic conditions. Six nurses had received formal training in health promotion or patient education within the past 3 years. Table 1 summarizes the demographic and professional characteristics of the study participants (*n* = 17).

### 3.2. Identified Themes and Subthemes

Five overarching themes with multiple subthemes were identified through thematic analysis. These themes capture the core patterns of meaning across patient and nurse perspectives regarding nursing support for exercise adherence in chronic disease management. Table 2 summarizes the main themes and subthemes identified from the qualitative analysis, along with their descriptions.

**Figure 1:**
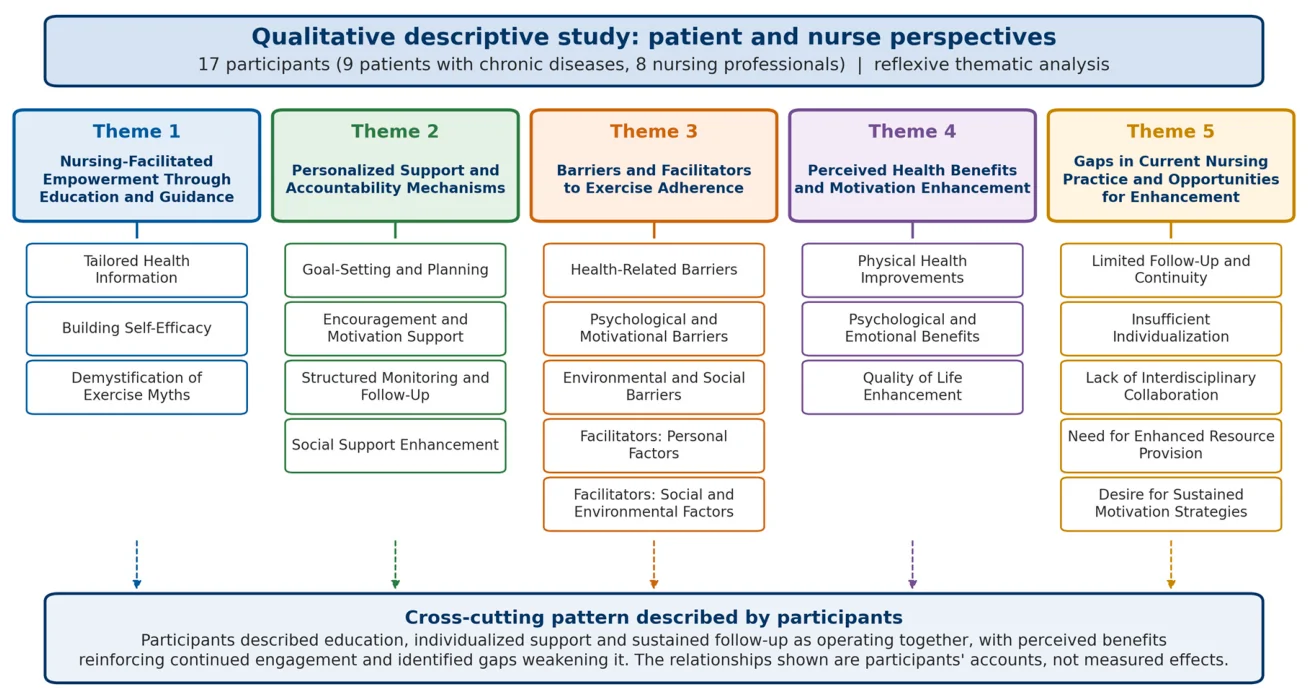
Thematic framework: nursing support for exercise adherence in chronic disease management. Five themes and their subthemes, constructed through reflexive thematic analysis [19] of interviews with 17 participants (nine patients, eight nurses). Theme and subtheme labels correspond verbatim to those in Table 2. The connections shown represent participants’ accounts of how these elements related to one another; they are not measured effects and imply no causal ordering.

The findings are organized within the thematic analysis framework (Figure 1), which outlines five main themes and their subthemes identified through reflexive thematic analysis of patient and nurse interviews.

### 3.3. Detailed Theme Development and Illustration

Table 3 sets out, for each of the strategies participants described, the operational detail they gave and the corresponding patient-reported experience, so that what nurses did and how patients received it can be read side by side. Illustrative extracts organised by theme follow.

#### 3.3.1. Theme 1: Nursing-Facilitated Empowerment Through Education and Guidance

Across both patient and nurse interviews, education and guidance emerged as central mechanisms through which nurses supported exercise engagement. This theme reflected a dynamic process where nurses imparted knowledge that enhanced patient understanding, increased confidence, and empowered autonomous decision-making.

##### Tailored Health Information

Patients consistently valued nurses who provided individualized, disease-specific explanations linking chronic disease pathophysiology with how exercise improved health outcomes. One patient with type 2 diabetes said:

“I was confused about how exercise helps diabetes. I thought my blood sugar could drop dangerously. The nurse explained how exercise helps my body use insulin better. Understanding the ‘why’ changed everything. I wasn’t just following orders; I understood I was taking care of myself.”

(Patient 3)

A patient with hypertension noted:

“Some doctors just say, ‘exercise is good,’ but [the nurse] explained how my blood pressure works and why movement helps. She tailored advice to my long work hours and we figured out realistic activity. That made sense to me.”

(Patient 7)

Nurses emphasized individualized education:

“I could give all patients the same pamphlet, but that won’t work. People need to understand why exercise matters for them. When they see the connection, they become motivated from within.”

(Nurse 4)

##### Building Self-Efficacy

Nurses supported patients’ confidence to exercise despite chronic disease limitations through reassurance, gradual goal-setting, and skill-building. One patient shared:

“I had tried to exercise before but overdid it and felt worse. The nurse helped me start with very small movements. Over weeks we increased gradually. Now I walk 45 minutes four times a week. She helped me believe I could do this.”

(Patient 2)

Nurses described strategies to build self-efficacy:

“I’m careful with my words. Instead of ‘You should walk 30 minutes,’ I say ‘Let’s start with 15 minutes, which is beneficial.’ I give specific feedback: ‘Maintaining this for four weeks shows real commitment.’ This builds confidence.”

(Nurse 2)

##### Demystification of Exercise Myths

Nurses actively corrected misconceptions and fears about exercise risks. Three misconceptions recurred across the patient accounts: that exertion would provoke dangerous hypoglycemia in diabetes, that it could trigger a stroke or a cardiac event, and that exercise-related fatigue or breathlessness signalled harm rather than normal physiological adaptation. Nurses described addressing these by naming the specific fear, offering evidence relating to the patient’s own condition, and reframing inactivity as carrying the greater risk. One patient recalled:

“I was terrified exercise would cause a stroke. My nurse explained that not exercising posed higher risk and showed studies. Knowing the facts helped me overcome fear.”

(Patient 5)

Nurses described this role:

“Many patients believe exercise is dangerous; I correct this with evidence. When they understand exercise is medicine, not danger, everything shifts.”

(Nurse 6)

#### 3.3.2. Theme 2: Personalized Support and Accountability Mechanisms

While education provided the knowledge foundation, patients highlighted ongoing personalized support, including goal-setting, encouragement, structured monitoring, and accountability.

##### Goal-Setting and Planning

Patients valued nurses who set realistic, meaningful goals collaboratively:

“The nurse asked what I wanted to achieve. I said I wanted to play with my grandchildren without getting out of breath. That became our goal. We built endurance with walking and tracked progress weekly. It made it personal and meaningful.”

(Patient 1)

Nurses described strategies:

“I help patients set goals that matter to them. We break larger goals into steps and review progress weekly.”

(Nurse 1)

##### Encouragement and Motivation Support

Patients emphasized ongoing reinforcement:

“After a few weeks, the novelty wore off. My nurse asked about my exercise, and when I said I was consistent, she seemed proud. Knowing someone cared kept me motivated.”

(Patient 4)

Nurses described delivering this recognition within routine follow-up consultations rather than through separate contacts:

“I celebrate every success, even small ones. I also use gentle accountability: ‘You said you’d try walking three times this week. How did it go?’ Most people try harder when asked.”

(Nurse 3)

##### Structured Monitoring and Follow-Up

Patients with regular follow-up reported better adherence:

“My nurse sets follow-up appointments and asks about my activity. That structure helps me stay accountable.”

(Patient 6)

Patients without consistent follow-up experienced decline:

“My nurse was supportive at first, then months passed without check-ins. I lost motivation and felt bad admitting it.”

(Patient 8)

Nurses acknowledged barriers:

“I try to follow up regularly, but as my caseload grows, structured follow-up sometimes falls away. Patients with consistent contact do best.”

(Nurse 5)

##### Social Support Enhancement

Nurses engaged patients’ families:

“My nurse talked to my family. Now my husband walks with me and my daughter sends encouraging texts. The nurse gave my family a role in my health.”

(Patient 9)

#### 3.3.3. Theme 3: Barriers and Facilitators to Exercise Adherence

##### Health-Related Barriers

Patients reported fatigue, pain, shortness of breath, and fears:

“Some days my blood sugar is unstable. I can’t exercise. My nurse helped me plan gentle movement on difficult days and more on good days.”

(Patient 3)

Nurses recognized these barriers:

“I don’t minimize limits. Patients with severe arthritis or heart disease do safe activities like water aerobics or chair exercises.”

(Nurse 4)

##### Psychological and Motivational Barriers

Patients noted motivation variability:

“Sometimes I’m motivated, sometimes not. My nurse acknowledges this and helps with strategies like tracking benefits or finding an exercise buddy.”

(Patient 7)

##### Environmental and Social Barriers

Patients faced facility, safety, time, and family challenges:

“No gym nearby and walking outside isn’t safe. My nurse helped me find free online videos and schedule realistic times.”

(Patient 2)

##### Facilitators: Personal Factors

Patients described tangible personal change as self-reinforcing:

“My energy improved in two weeks, I slept better and could fit into old clothes. That motivated me to keep going.” (Patient 1) Facilitators: Social and Environmental Factors. Patients also identified family involvement and the availability of affordable, nearby options as sustaining factors: “My wife walks with me most mornings. Family involvement is one of the biggest helps.”

(Patient 6)

#### 3.3.4. Theme 4: Perceived Health Benefits and Motivation Enhancement

##### Physical Health Improvements

Patients noted disease control and functional gains:

“My blood sugar improved and medication was reduced. Knowing exercise helps my condition makes me take it seriously.”

(Patient 5)

Nurses reinforced improvements:

“I show lab improvements and connect them to exercise. Concrete evidence is motivating.”

(Nurse 2)

##### Psychological and Emotional Benefits

Patients reported mood and confidence gains:

“My mood improved, I have more energy and feel accomplished maintaining my routine.”

(Patient 4)

##### Quality of Life Enhancement

Patients described regained independence and life satisfaction:

“At 72, I can pick up grandchildren, garden, walk to neighbors. Being able to do what I love is quality of life. My nurse helped me achieve that.”

(Patient 9)

#### 3.3.5. Theme 5: Gaps in Current Nursing Practice and Opportunities for Enhancement

##### Limited Follow-Up and Continuity

Patients experienced declining support over time:

“After initial engagement, follow-up decreased. I needed it most to maintain motivation.”

(Patient 8)

Nurses cited systemic barriers:

“We see too many patients with too little time. Acute problems take priority over exercise support.”

(Nurse 5)

##### Insufficient Individualization

Patients received generic advice:

“I was just told to ‘exercise regularly.’ Better support would help find activities that fit my life.”

(Patient 2)

Nurses acknowledged time constraints:

“Individualized care takes time, and I don’t always have that luxury.”

(Nurse 1)

##### Lack of Interdisciplinary Collaboration

Patients noted fragmented care:

“My nurse recommended exercise, but my doctor prescribed medications causing fatigue. Better coordination would help.”

(Patient 7)

Nurses recognized siloed practice:

“Ideally, a multidisciplinary team would coordinate around goals. Currently, we work in silos.”

(Nurse 6)

##### Need for Enhanced Resource Provision

Patients wanted tangible resources:

“A written guide or video for home exercises would increase my confidence.”

(Patient 3)

Nurses confirmed value:

“A library of videos or handouts would reinforce our conversations and support continuity.”

(Nurse 4)

##### Desire for Sustained Motivation Strategies

Patients wanted long-term strategies:

“Motivation dips over time. I need help to rekindle it, like reviewing benefits or connecting with others.”

(Patient 9)

Across both groups, nursing support through education, personalized guidance, and structured follow-up was described as helping patients engage in regular exercise despite chronic disease. Patients valued individualized information, confidence-building, correction of misconceptions, goal-setting, encouragement, and family involvement. Perceived physical, psychological, and functional benefits were described as reinforcing sustained engagement. Limited follow-up, insufficient individualisation, absent interdisciplinary coordination, and scarce resources were identified as the principal shortfalls. Overall, participants described sustained, personalized nursing support as central to how they engaged with exercise. Because no measure of exercise behavior or clinical outcome was collected, these are perceived rather than demonstrated effects. The nursing group spanned diploma to master’s preparation and responses were not analyzed by qualification level; the implications of that sample composition are considered in Section 4.5 and Section 4.9.

## 4. Discussion

### 4.1. Summary of Key Findings

This study explored nursing support for exercise adherence in chronic disease patients through the dual perspectives of patients and nurses, identifying five overarching themes that illuminate the complex dynamics underlying sustainable behavior change. The accounts indicate that nursing support experienced as effective extends beyond information provision, combining educational, motivational, relational, and structural elements that participants described as needing to be sustained over time. These are participants’ perceptions of what helped; the study did not measure whether behavior changed.

### 4.2. Theoretical and Conceptual Integration

The study’s findings align with and extend current theoretical understanding of behavior change in several important ways.

Self-Determination Theory. Self-Determination Theory posits that sustained motivation for behavior change emerges when individuals experience satisfaction of three fundamental psychological needs: competence (feeling effective and capable), autonomy (having choice and control over one’s actions), and relatedness (feeling supported and connected to others). The present study’s findings map closely onto these theoretical constructs [15]. Theme 1, concerning nursing-facilitated education and empowerment, maps onto competence: participants described increased understanding of exercise mechanisms and building confidence in exercise capability. Theme 2, addressing personalized goal-setting and collaborative planning, maps onto autonomy, in that participants described exercise decisions as reflecting patient values and preferences rather than imposed medical directives. Theme 2’s emphasis on ongoing encouragement and the nurse–patient relationship supported relatedness, with patients experiencing nursing support and recognition of their efforts as genuinely motivating.

Conversely, Theme 5’s identified gaps, particularly insufficient individualization and limited follow-up, can be conceptualized as undermining these psychological needs. When nurses prescribed generic recommendations without eliciting patient input, autonomy was diminished. When follow-up ceased, relatedness decreased and patients lost the external reinforcement of nurse recognition and support necessary to maintain intrinsic motivation.

Social Cognitive Theory. Social Cognitive Theory [14] emphasizes the reciprocal relationships among personal factors (including self-efficacy and outcome expectations), environmental factors, and behavior. The present findings illustrate how nursing interventions influenced all three elements. Nurses built self-efficacy through graduated goal-setting and positive feedback (personal factors). Nurses helped patients develop positive outcome expectations by connecting exercise to meaningful personal goals and helping them experience health benefits (personal factors and outcome expectations). Nurses also modified environmental factors by facilitating family involvement, identifying accessible exercise options, and providing structure and accountability.

Self-Efficacy and Pender’s Model. Bandura’s self-efficacy construct provides a further lens: the graduated goal-setting, positive reinforcement, and myth-correction strategies described by both patients and nurses directly address the sources of self-efficacy identified by Bandura (mastery experiences, vicarious reinforcement, verbal persuasion, and physiological feedback). The alignment between these strategies and SCT predictions reinforces the theoretical basis for structured nursing-led exercise support. Pender’s Health Promotion Model is equally instructive: the model predicts that perceived benefits, perceived barriers, and interpersonal influences are the most proximal determinants of health-promoting behavior. Theme 3’s identification of barriers (fear, fatigue, lack of facilities) and Theme 2’s emphasis on personalized encouragement map directly onto Pender’s barrier-reduction and interpersonal influence pathways. Nurses who explicitly targeted these cognitions, correcting fears, facilitating social support, and reinforcing observable health gains, described enacting the mechanisms that Pender’s model identifies as proximal to health-promoting behavior [16]. Whether doing so alters measured activity was not examined here and cannot be inferred from these data.

### 4.3. Contributions to Knowledge and Practice

This research contributes to nursing science and practice in several significant ways.

Empirical Contribution. This study provides rich, detailed empirical evidence regarding how nursing support is experienced and perceived by patients in real-world chronic disease management contexts. Few previous studies have examined the patient’s experience of nursing support for physical activity, and fewer still have simultaneously examined nurse perspectives on their role. This dual-perspective approach illuminates not only what nurses do but how patients experience those interventions and what they perceive as most influential. The finding that personalized, ongoing support matters more than one-time education challenges the notion that brief health education interventions are sufficient for sustained behavior change.

Theoretical Contribution. The study extends existing behavior change theories by highlighting the specific mechanisms through which nursing support facilitates psychological need satisfaction (competence, autonomy, relatedness) and how these psychological needs, once satisfied, fuel intrinsic motivation for sustained exercise engagement. The integrated model emerging from these findings emphasizes that sustainable behavior change requires not isolated interventions but rather a coherent system of support addressing knowledge gaps, building skills and confidence, respecting autonomy and individual circumstances, providing ongoing relational support, and creating accountability structures.

Practice Contribution. The findings have direct implications for how nurses should approach exercise promotion in chronic disease contexts. Rather than viewing exercise promotion as a minor component of nursing care addressed through brief discussions or generic education, the findings suggest that exercise promotion requires dedicated time, systematic planning, ongoing monitoring, and integration across the nurse–patient relationship. The identification of specific gaps in current practice (limited follow-up, insufficient individualization, lack of interdisciplinary collaboration) provides clear targets for practice improvement.

### 4.4. Alignment with Existing Literature

The present findings align with and meaningfully extend the existing literature on exercise adherence and nurse-led health promotion. Sluijs et al. [23] identified supervision, goal-setting, and patients’ beliefs about exercise as the primary correlates of exercise compliance in clinical settings, all of which emerge prominently in Themes 1 and 2 of the present study. The centrality of self-efficacy as a mechanism of adherence is consistent with Karataş and Polat [18], who demonstrated in a nurse-led exercise program using Pender’s Health Promotion Model that nurses’ systematic effort to build patient confidence produced significantly improved exercise behavior in coronary artery patients. Our findings provide qualitative texture to this quantitative finding: patients described self-efficacy as built through graduated goals, reassurance, and myth-correction, mechanisms that quantitative studies cannot capture in depth. The importance of personalized, individualized support identified here aligns with Sha and Pi [6], whose randomized controlled trial demonstrated that narrative nursing (a form of individualized relational engagement) significantly improved treatment adherence and self-management in patients with diabetes. Our qualitative data illuminate why this works: individualized support satisfies patients’ need for autonomy and relatedness, which in turn drives intrinsic motivation (consistent with SDT [15]). The theme of barriers and facilitators to exercise adherence (Theme 3) is consistent with Spiteri et al. [7], whose systematic review identified motivational fluctuation, social support, and health-related limitations as the most frequently cited determinants of physical activity participation, the same three categories that dominate our patient interviews. However, the present study extends these findings by demonstrating specifically how nursing interventions can modify each category: correcting fear-based beliefs (addressing health-related barriers), facilitating family involvement (strengthening social support), and providing ongoing motivational check-ins (addressing fluctuation). Resnicow et al. [24] demonstrated that motivational interviewing improves health-promoting behavior change; our findings support this by showing that the same relational mechanisms—patient-directed dialogue, barrier acknowledgement, and collaborative problem-solving—are the strategies nurses describe as most effective for exercise promotion. Together, these comparisons indicate that the present accounts are consistent with the broader evidence base and add practice-relevant detail about how nursing support is experienced in real-world chronic disease settings. Because the comparator studies measured behavioral outcomes and the present study did not, the convergence concerns the mechanisms described rather than the effects obtained.

### 4.5. Novel Findings and Insights

While many findings align with existing literature, some novel insights emerge from this research. The most distinctive contribution of this study lies in Theme 5 (Gaps in Current Nursing Practice and Opportunities for Enhancement), which documents, from both nurse and patient perspectives, the structural and systemic barriers that prevent nurses from delivering comprehensive exercise support even when they possess the knowledge and motivation to do so. This moves the analysis beyond a catalog of patient opinions toward a theoretically and practically grounded account of how healthcare system architecture shapes nursing behavior and patient outcomes. Some nurses’ accounts offered a tentative impression of possible differences between government and private facility contexts: nurses working in government tertiary hospitals more often described high patient caseloads, competing acute-care priorities, and limited interdisciplinary coordination as barriers to sustained exercise support, whereas nurses in private clinics did not raise these concerns to the same extent. This comparison was not a pre-specified focus of data collection or analysis, and the study was not designed or powered to compare facility types systematically; the observation is therefore best regarded as hypothesis-generating rather than a confirmed finding. If corroborated by future research designed to compare facility types directly, this pattern could have implications for resource allocation and organizational support for exercise promotion; however, firm conclusions about facility-level disparities cannot be drawn from the present data.

Nurse preparation and capability for complex behavior-change work. The nurse sample spanned diploma to master’s preparation, and the strategies participants described differ considerably in the preparation they require. Providing condition-specific explanation, correcting a stated misconception, and scheduling a review appointment sit within generalist practice. Stage-matched behavioral counselling and motivational-interviewing-style dialogue are, by contrast, structured techniques with defined competencies that require dedicated training and supervised practice, and six of the eight nurses had received formal training in health promotion or patient education within the preceding three years. Responses were not analyzed by qualification level and the sample was not designed to support such a comparison, so no claim is made that qualification determined which strategies a nurse described. The distribution does, however, indicate that a uniform capability for complex behavior-change work should not be assumed across a mixed-qualification nursing workforce, and that access to structured training is a plausible determinant of what individual nurses are able to offer. This has direct implications for how exercise-promotion protocols are specified: a protocol that presumes motivational interviewing competence will be implemented unevenly unless the corresponding training is resourced.

The Motivation Sustainability Paradox. Both patients and nurses recognized that exercise motivation is not stable over time but naturally fluctuates. Interestingly, rather than attempting to maintain constant high motivation, more effective support involved helping patients navigate through periods of lower motivation using strategies like reviewing benefits achieved, setting new goals, connecting with others doing similar things, and returning to foundational discussions about personal motivation. This suggests that nursing support for exercise adherence should explicitly incorporate strategies for maintaining adherence during inevitable periods of lower motivation.

The “Demystification” Function of Education. While health education for exercise is well-established in nursing literature, the specific function of correcting fear-based misconceptions, particularly the fear that exercise could exacerbate chronic disease, emerged as unexpectedly powerful. Many participants harbored significant fear and misconceptions about exercise safety in chronic disease contexts. Nursing education that explicitly addressed these fears with evidence-based reassurance appeared to be a critical gating factor for even initiating exercise behavior. This suggests that preemptively addressing common fears and misconceptions about exercise in chronic disease may be particularly important.

The Structural Role of Scheduled Follow-Up. While follow-up is mentioned in nursing literature, the specific finding that patients responded to scheduled, structured follow-up appointments dedicated to exercise monitoring emerged strongly. Several patients described how knowing when their next exercise-specific check-in would occur created accountability and facilitated adherence between appointments. This suggests that incorporating scheduled, regular exercise-focused follow-up appointments into clinical protocols may be more effective than simply offering follow-up “as needed.”

### 4.6. Implications for Nursing Practice

The findings highlight important implications for nursing practice. Exercise promotion should be embedded as a core nursing responsibility, comparable to medication management and clinical monitoring, and systematically integrated into nursing care plans with dedicated time for delivery. To support consistent implementation, healthcare systems should adopt standardized exercise promotion protocols that provide clear guidance while allowing flexibility for individualization. For instance, protocols may require early exercise education for patients with chronic conditions, with structured follow-up, while enabling nurses to tailor content to patients’ needs, preferences, and functional capacity.

Nursing practice should also shift from ad hoc discussions of exercise to structured and proactive follow-up. Regularly scheduled follow-up focused on exercise adherence is recommended, with more frequent contact for patients at higher risk of non-adherence. Investment in accessible patient education resources, such as written materials, instructional videos, and referrals to structured exercise programs, would further support nurse-led interventions. Effective exercise promotion additionally relies on interdisciplinary collaboration to address barriers such as medication-related limitations. Finally, nurses require ongoing education in exercise prescription, behavior change strategies, and motivational interviewing, alongside organizational support that ensures sufficient time and resources to deliver comprehensive exercise promotion.

### 4.7. Implications for Healthcare Systems and Policy

Beyond individual nursing practice, the findings indicate important system-level implications. Healthcare organizations should allocate adequate resources to support exercise promotion, including protected nursing time, patient education materials, access to structured exercise programs, and mechanisms for interdisciplinary coordination. Without sufficient resourcing, comprehensive exercise promotion is unlikely to be implemented consistently or sustainably.

System-wide accountability structures should also recognize exercise promotion as a component of quality care. Performance metrics should incorporate indicators related to exercise promotion and adherence support, reinforcing its value within nursing practice. Interprofessional education programs should prioritize training in health behavior change and evidence-based exercise promotion strategies across disciplines. In addition, supportive healthcare policies are needed to enable implementation, including policies that allow adequate nursing time, facilitate coverage for exercise-related interventions, and strengthen community-level infrastructure that supports physical activity.

### 4.8. Strengths of the Study

This study has several strengths. The dual-perspective design, incorporating both patient and nurse viewpoints, allows convergence and divergence between the two accounts to be examined within a single study. Rigorous qualitative methods were applied, including a descriptive design with reflexive thematic analysis, member checking, peer debriefing, and reflexive documentation. Semi-structured interviews generated detailed accounts, and the patient sample varied by chronic condition, age, activity level, and educational background. Grounding in a defined healthcare setting allowed organizational and cultural context to be considered, and the analytic process is documented in sufficient detail for readers to assess how the themes were constructed.

### 4.9. Limitations

Several limitations bear directly on how these findings should be read. First, and most consequential, the study did not measure exercise behavior. No data were collected on the frequency, intensity, duration, or regularity of participants’ activity, and no clinical or biochemical outcomes were recorded. Every statement in this report about benefit, improvement, or effectiveness is a participant’s perception. None of them constitutes evidence that nursing support altered activity levels or health outcomes, and the study is not capable of supporting such a claim.

Second, the sample of 17 is appropriate for qualitative description but small, drawn from one urban region, and not intended to support generalization. The findings reflect the organizational, cultural, and resource context of five institutions in the Riyadh region and may not transfer to rural or other regional settings.

Third, the patient sample was dominated by type 2 diabetes (six of nine) and hypertension (five of nine), with one participant reporting cardiovascular disease and none reporting chronic obstructive pulmonary disease, malignancy, or inflammatory arthritis. The barriers described, and therefore the nursing responses to them, are likely to differ for conditions involving other functional limitations, such as severe chronic obstructive pulmonary disease, advanced malignancy, or significant joint disease, where exertional breathlessness, treatment-related fatigue, or pain constrain activity in ways the present participants did not describe. Transferability to those populations should not be assumed.

Fourth, recruitment relied on voluntary response to clinic-based invitation supplemented by snowball sampling, which is likely to have favored participation by patients and nurses already engaged with exercise promotion. The probable direction of this bias can be stated: patients who disengaged from services, who found nursing support unhelpful, or who abandoned exercise altogether are the least likely to have volunteered. The perceived effectiveness reported in Themes 1, 2, and 4 is therefore likely to be an overestimate relative to the wider clinic population, and the shortfalls documented in Theme 5 are likely to be understated. Two individuals declined participation and, under the approved consent procedure, no information was collected from them, so this expected direction of bias cannot be checked against the accounts of non-participants.

Fifth, transcript length and depth varied between participants. All 17 transcripts were coded and each contributed to the thematic structure, but richness was not formally rated, and the more extended accounts supplied a greater share of the illustrative extracts presented.

Sixth, all nursing participants worked in direct clinical roles. Three held a master’s degree, but the perspectives of advanced practice nurses and clinical nurse specialists in dedicated chronic disease roles are not represented, and responses were not analyzed by qualification level. Data were collected at a single time point, precluding examination of change over time, and rest on self-reported accounts of clinical interactions rather than observation of those interactions. Interviews were conducted in Arabic and analyzed in English translation; although independent back-translation was used, some subtlety of expression is likely to have been lost.

### 4.10. Recommendations

The recommendations below follow from specific findings and are stated at the level of detail the data support. Each is a proposal for testing rather than a demonstrated effect.

For clinical practice, five changes follow most directly. Screening for fear-based beliefs at the point of first activity advice is warranted by Theme 1: participants described fear of hypoglycemia and of precipitating a cardiac or cerebrovascular event as blocking initiation, and described these fears as resolvable by a short, condition-specific conversation. A brief structured enquiry, asking what the patient believes exercise might do to their condition, would identify this at low cost. Second, the exercise goal should be recorded in the patient’s own functional terms rather than in guideline minutes; the goal participants engaged with was the activity they wanted to recover, not a weekly target. Third, the initial prescription should be set deliberately below the guideline threshold and escalated, since the patient who described sustained activity had previously abandoned it after over-exertion. Fourth, exercise-specific review should be scheduled at named intervals rather than offered as needed, because participants attributed adherence to the existence of a booked review and attributed decline to its lapse. Fifth, where a household member is available and the patient consents, that person should be given an explicit role, which participants described as among the strongest supports.

For nursing education, the distinction drawn in Section 4.5 matters. Condition-specific explanation and structured follow-up can reasonably be expected of all registered nurses; motivational interviewing and stage-matched behavioral counselling are defined competencies requiring dedicated training and supervised practice, and protocols that presume them without resourcing them will be applied unevenly across a mixed-qualification workforce.

For services and policy, the constraints nurses described were structural rather than attitudinal: caseload, the precedence of acute problems, the absence of shared patient resources, and the absence of a referral pathway to physiotherapy or exercise services. Protected time for exercise review, a shared institutional library of written and video patient materials, and a defined referral route address these directly and are measurable as implementation targets.

For research, the most useful next step is the one this study cannot take. A prospective study pairing the support components identified here with objective activity measurement, such as accelerometry or validated activity logging, alongside routine clinical indicators, would establish whether the mechanisms participants described translate into behavior change. Testing whether scheduled exercise-specific review outperforms as-needed follow-up is a discrete and feasible question arising directly from Theme 2. Replication in conditions other than diabetes and hypertension, and in rural settings, would address the transferability limits set out above.

## 5. Conclusions

Exercise adherence remains a difficult component of chronic disease management. In this qualitative study of nine patients and eight nurses in the Riyadh region, both groups described nursing support for physical activity as extending well beyond information provision, encompassing condition-specific explanation, correction of fear-based beliefs, goal-setting framed in the patient’s own terms, graduated escalation, scheduled review, and involvement of the family. Both groups also identified where that support fell short: follow-up that lapsed under caseload pressure, advice that remained generic, limited coordination with medical and allied colleagues, and an absence of tangible patient resources. Notably, nurses attributed these shortfalls to structural constraints rather than to a lack of knowledge or willingness, and patients described the consequences in terms that corresponded closely.

These are participants’ accounts within one urban Saudi setting. The study measured no activity and no clinical outcome, and its findings therefore identify what patients and nurses regard as helpful, not what demonstrably works. Whether the components described here change exercise behavior is a question for prospective studies that pair them with objective activity measurement, and the specific proposition that scheduled exercise-specific review outperforms as-needed follow-up is the most immediately testable of them. What the present findings support is a more modest and still useful claim: that positioning exercise promotion as a defined nursing responsibility, with the protected time, shared resources, and referral pathways that nurses identified as missing, addresses constraints that both parties to the clinical encounter independently described.

## Figures and Tables

**Table 1:** Participant Characteristics (*n* = 17).

Characteristic	Patient Group (*n* = 9)	Nurse Group (*n* = 8)
Age (years)		
Mean ± SD	54.2 ± 11.8	42.1 ± 10.4
Range	38–72	29–58
Gender		
Female, n (%)	5 (55.6%)	6 (75.0%)
Male, n (%)	4 (44.4%)	2 (25.0%)
Chronic Disease Type		
Type 2 Diabetes Mellitus	6 (66.7%)	—
Hypertension	5 (55.6%)	—
Cardiovascular Disease	1 (11.1%)	—
Multiple Chronic Conditions	3 (33.3%)	—
Disease Duration (years)		
Mean ± SD	8.4 ± 5.2	—
Range	2–18	—
Current Physical Activity Level		
Low	3 (33.3%)	—
Moderate	4 (44.4%)	—
High	2 (22.2%)	—
Educational Level		
Primary/Secondary	4 (44.4%)	—
High School	3 (33.3%)	—
University	2 (22.2%)	—
Nursing Diploma	—	3 (37.5%)
Bachelor’s Degree	—	2 (25.0%)
Master’s Degree	—	3 (37.5%)
Employment Status		
Employed	4 (44.4%)	8 (100%)
Retired/Unemployed	5 (55.6%)	—
Years of Experience		
Mean ± SD	—	13.8 ± 8.2
Range	—	4–28
Formal Health Promotion Training		
Yes	—	6 (75.0%)
No	—	2 (25.0%)

Note: Chronic disease categories are not mutually exclusive; patients with more than one condition are counted in each applicable disease-specific row as well as in the “Multiple Chronic Conditions” row, so totals exceed *n* = 9.

**Table 2:** Themes and Subthemes with Descriptions.

Theme	Subtheme	Description
Theme 1: Nursing-Facilitated Empowerment Through Education and Guidance	Tailored Health Information	Nurses provided personalized information about disease mechanisms, exercise benefits, and appropriate activity types, increasing patient understanding and confidence
	Building Self-Efficacy	Nursing interventions explicitly focused on increasing patients’ confidence in their ability to successfully exercise despite chronic disease constraints
	Demystification of Exercise Myths	Nurses corrected misconceptions about exercise dangers in chronic disease populations, reducing fear-based barriers
Theme 2: Personalized Support and Accountability Mechanisms	Goal-Setting and Planning	Collaborative development of realistic, individualized exercise goals with concrete action steps
	Encouragement and Motivation Support	Ongoing positive reinforcement, celebration of achievements, and sustenance of motivation over time
	Structured Monitoring and Follow-Up	Regular assessment of exercise progress, problem-solving around adherence challenges, and adjustment of plans as needed
	Social Support Enhancement	Facilitation of family involvement and peer support for exercise engagement
Theme 3: Barriers and Facilitators to Exercise Adherence	Health-Related Barriers	Physical limitations, fear of symptom exacerbation, fatigue, and disease-related complications
	Psychological and Motivational Barriers	Variability in motivation, low mood, loss of interest, and perception that lifestyle change is overwhelming
	Environmental and Social Barriers	Lack of accessible exercise facilities, safety concerns, competing time demands, and insufficient family support
	Facilitators: Personal Factors	Individual determination, perceived health benefits, improved energy levels, and quality of life improvements
	Facilitators: Social and Environmental Factors	Family encouragement, exercise facilities proximity, affordable options, and structured group programs
Theme 4: Perceived Health Benefits and Motivation Enhancement	Physical Health Improvements	Improved blood glucose control, reduced blood pressure, increased strength and endurance, and weight management
	Psychological and Emotional Benefits	Improved mood, reduced anxiety, enhanced self-confidence, and increased life satisfaction
	Quality of Life Enhancement	Improved functional capacity, increased independence, enhanced social engagement, and greater overall life satisfaction
Theme 5: Gaps in Current Nursing Practice and Opportunities for Enhancement	Limited Follow-Up and Continuity	Inconsistent or insufficient follow-up communication, inadequate monitoring of long-term adherence, and lack of continuity across care transitions
	Insufficient Individualization	Generic exercise advice rather than truly personalized plans accounting for individual preferences, constraints, and goals
	Lack of Interdisciplinary Collaboration	Limited coordination between nursing, medical providers, physiotherapists, and psychological support services
	Need for Enhanced Resource Provision	Inadequate provision of written materials, exercise demonstrations, or access to exercise programs
	Desire for Sustained Motivation Strategies	Recognition that motivation requires ongoing nurturing beyond initial discussions

**Table 3:** Operationalised nursing strategies described by participants, with corresponding patient-reported experiences.

Nursing Strategy (Theme/Subtheme)	Operational Detail as Described by Participants	Corresponding Patient-Reported Experience
Condition-specific explanation (T1: Tailored Health Information)	Explaining, in terms specific to the patient’s own condition, how activity affects that condition, in place of issuing a standard pamphlet (Nurse 4); adjusting the recommendation to the patient’s working hours (Patient 7).	Understanding the reason for the advice shifted one patient from following instructions to self-directed care (Patient 3); advice matched to long working hours was described as usable (Patient 7).
Graduated goal escalation (T1: Building Self-Efficacy)	Opening with a target below the guideline figure (“Let’s start with 15 min”) and giving effort-focused rather than outcome-focused feedback (Nurse 2).	A patient who had previously over-exerted and stopped rebuilt to walking 45 min four times weekly and described a change in what she believed she could do (Patient 2).
Correction of fear-based beliefs (T1: Demystification of Exercise Myths)	Naming the specific fear, presenting condition-specific evidence, and reframing inactivity as carrying the greater risk (Nurse 6).	Fear that exertion would provoke a stroke was resolved and activity was initiated (Patient 5).
Elicitation of a personally meaningful goal (T2: Goal-Setting and Planning)	Asking what the patient wants to be able to do, then decomposing that into steps reviewed weekly (Nurse 1).	Playing with grandchildren without breathlessness became the stated goal, with weekly progress tracking; the patient described the goal as personal and meaningful (Patient 1).
Recognition and gentle accountability (T2: Encouragement and Motivation Support)	Marking small successes and referring back to the patient’s own stated intention during routine follow-up consultations (“You said you’d try walking three times this week. How did it go?”) (Nurse 3).	Being asked about activity, and being seen to have kept to it, sustained motivation once initial novelty had faded (Patient 4).
Scheduled exercise-specific review (T2: Structured Monitoring and Follow-Up)	Booking follow-up appointments at which activity is asked about; nurses reported this lapsing as caseload rises (Nurse 5).	Patients with scheduled review described the structure itself as what kept them accountable (Patient 6); a patient whose review lapsed lost motivation and was reluctant to disclose it (Patient 8).
Recruitment of the family (T2: Social Support Enhancement)	Speaking directly with family members and giving them a defined role in the plan.	A husband became a walking partner and a daughter sent encouraging messages (Patient 9); spousal participation was described as among the strongest supports (Patient 6).
Adaptation to fluctuating clinical status (T3: Health-Related Barriers)	Planning lighter activity for unstable days and more for good days; substituting water aerobics or seated exercise where joint or cardiac limits apply (Nurse 4).	Days of unstable blood glucose no longer meant abandoning activity altogether (Patient 3).
Substitution where facilities are absent (T3: Environmental and Social Barriers)	Identifying free home-based video resources and fixing realistic times for their use.	A patient with no nearby facility and no safe walking route continued exercising at home (Patient 2).
Feedback of objective change (T4: Physical Health Improvements)	Showing laboratory results and connecting them explicitly to reported activity (Nurse 2).	Improved blood glucose and a medication reduction increased the seriousness with which the patient treated exercise (Patient 5).

## Data Availability

The datasets collected and analyzed during the current study are not publicly available. The data consist of individual interview transcripts that contain contextual detail capable of identifying participants, and the terms of the ethical approval and of participant consent do not permit their public deposition. De-identified excerpts supporting the reported themes are available from the corresponding author on reasonable request, subject to approval by the King Saud University Institutional Review Board.

## Supplementary Materials

The following supporting information can be downloaded at https://www.mdpi.com/article/10.3390/healthcare14182980/s1. Table S1: Completed COREQ (Consolidated Criteria for Reporting Qualitative Research) 32-item checklist. Table S2: Patient and nurse semi-structured interview guides.
