# Supplementary material for: Nursing Support for Exercise Adherence in Chronic Disease Management: A Qualitative Descriptive Study of Patient and Nurse Perceptions
Source: Healthcare (Basel). 2026 Sep 12;14(18):2980. doi: 10.3390/healthcare14182980 (PMC13606859; doi:10.3390/healthcare14182980)
Supplement: Supplementary file 1 [file healthcare-14-02980-s001.zip › healthcare-4473898-supplementary.pdf]

COREQ Checklist (Supplementary Table S1)

Consolidated criteria for reporting qualitative research (Tong, Sainsbury and Craig, 2007). Item locations refer to the revised manuscript. Where an item was not performed, this is stated rather than left blank.

Domain 1: Research team and reflexivity

Personal characteristics

#	Item	Response	Location
1	Interviewer/facilitator	Interviews were led by A.M.A. Interviews were conducted by trained research team members with prior qualitative research experience.	2.3
2	Credentials	The interviewer holds a PhD. The lead researcher is a nurse with 15 years of clinical and 8 years of research experience.	2.3, 2.4
3	Occupation	Academic nursing and exercise physiology faculty, King Saud University.	Title page, 2.4
4	Gender	Academic nursing and exercise physiology faculty, King Saud University..	Title page, 2.4
5	Experience and training	The lead researcher has 8 years of research experience and substantial knowledge of nursing practice, chronic disease management, and health behaviour change; interviewers had prior qualitative research experience.	2.3, 2.4

Relationship with participants

#	Item	Response	Location
6	Relationship established	No. The interviewer had no prior relationship with any participant before the study.	2.3
7	Participant knowledge of the interviewer	Participants received detailed study information and an information sheet before consent, covering study purposes, procedures, risks, benefits, and rights.	2.2.2, 2.7
8	Interviewer characteristics	Reported. The lead researcher's clinical nursing background, its value for understanding both perspectives, and the need to bracket assumptions about "ideal" nursing practice are set out, along with the reflexive journal kept throughout and the team's explicit discussion of professional backgrounds, personal experiences with exercise, and beliefs about nursing's role in health promotion.	2.4

Domain 2: Study design

Theoretical framework

#	Item	Response	Location
9	Methodological orientation and theory	Qualitative descriptive design with reflexive thematic analysis (Braun and Clarke). Three frameworks (Social Cognitive Theory, Self-Determination Theory, Pender's Health Promotion Model) oriented the interview guide and interpretation; the manuscript states explicitly that they were not used to pre-code the data.	1.2, 2.1, 2.5

Participant selection

#	Item	Response	Location
10	Sampling	Purposive sampling, with maximum-variation sampling for the patient group, supplemented by snowball sampling. Rationale for combining the two is given.	2.2.2
11	Method of approach	Recruitment through chronic disease outpatient clinics using flyers, information sheets, and verbal announcements, supplemented by social media and professional networking channels, and by snowball sampling.	2.2.2
12	Sample size	17 participants: 9 patients with chronic diseases and 8 nursing professionals. Justified by information power (Malterud et al.) rather than by a numeric rule.	2.2.2, 3.1
13	Non-participation	Two individuals approached declined. No demographic, clinical, or experiential information was collected from them, consistent with the approved consent procedure; the consequence for interpretation is stated in the Limitations.	2.7, 4.9

Setting

#	Item	Response	Location
14	Setting of data collection	A quiet, private location chosen by each participant, or a secure online platform.	2.3
15	Presence of non-participants	No one other than the participant and the interviewer was present.	2.3
16	Description of sample	Full demographic and professional characteristics for both groups are reported in the text and in Table 1: age, gender, chronic disease type, disease duration, current activity level, educational level, employment status, years of nursing experience, and formal health promotion training.	3.1, Table 1

Data collection

#	Item	Response	Location
17	Interview guide	Semi-structured guides developed by the research team and informed by existing literature; content areas listed for both the patient guide and the nurse guide, with representative verbatim items quoted for each. Complete guides supplied as Supplementary Table S2 and available from the corresponding author.	2.3, Table S2
18	Repeat interviews	No. No repeat interviews were conducted.	2.3
19	Audio/visual recording	All interviews were audio-recorded with participant consent and transcribed verbatim in Arabic.	2.3
20	Field notes	Not reported as a formal procedure. A reflexive journal was kept by the lead researcher throughout data collection and analysis, and analytic memos were recorded in NVivo during coding.	2.4, 2.5
21	Duration	30 to 60 minutes, mean 45 minutes, across the whole sample. Durations are not disaggregated by participant group.	2.3
22	Data saturation	Informational redundancy was reached after 15 interviews; the final two participants confirmed rather than extended the established thematic structure.	2.2.2
23	Transcripts returned	Yes, in part. Two transcripts were returned to participants for review and comment on their accuracy before analysis. This is distinct from the member checking of preliminary findings recorded under item 28.	2.6

Domain 3: Analysis and findings

Data analysis

#	Item	Response	Location
24	Number of data coders	Multiple. Initial coding was conducted independently by multiple team members, who then compared and discussed coding decisions; each researcher created coding nodes and memos within a shared NVivo project.	2.4, 2.5
25	Description of the coding tree	Coding is described narratively through the six phases of reflexive thematic analysis, consistent with that approach. Both semantic and latent codes were generated, with worked examples given. The resulting theme and subtheme structure is presented in full in Table 2 and Figure 1. No separate coding-tree diagram was produced.	2.5, Table 2, Figure 1
26	Derivation of themes	Themes were derived from the data. Coding was data-driven rather than predetermined, with codes generated from the explicit content of participant responses; candidate themes	2.5

#	Item	Response	Location
		were developed in Phase 3 and refined across the full dataset in Phase 4.	
27	Software	NVivo 14, used for data management, coding, and retrieval only. The manuscript states that all interpretive and thematic decisions were made by the research team through iterative discussion rather than by software.	2.5
28	Participant checking	Yes. Preliminary findings were shared with a purposively selected subset of participants (4 patients, 3 nurses) for feedback on interpretive accuracy.	2.6

Reporting

#	Item	Response	Location
29	Quotations presented	Yes. Verbatim quotations are presented throughout Section 3.3 for every subtheme, identified by participant number (Patient 1-9, Nurse 1-6). Quotations are additionally mapped to nursing strategies and patient-reported experiences in Table 3.	3.3, Table 3
30	Data and findings consistent	Yes. Each theme and subtheme is illustrated with supporting extracts from both participant groups where both contributed. Theme and subtheme labels are identical across Table 2, Figure 1, Table 3, and the Section 3.3 narrative. Interpretive claims are qualified throughout as participants' accounts rather than measured effects.	3.2, 3.3, 4.1
31	Clarity of major themes	Yes. Five themes are presented, each with a description in Table 2, a diagram in Figure 1, and a narrative section with extracts in Section 3.3.	3.2, 3.3, Table 2, Figure 1
32	Clarity of minor themes	Yes. Twenty subthemes are reported across the five themes, each defined in Table 2 and illustrated in Section 3.3. Divergent cases are presented, including patients whose follow-up lapsed and nurses reporting that structured follow-up falls away as caseload rises.	3.2, 3.3

SUPPLEMENTARY MATERIAL 2: INTERVIEW GUIDES (Supplementary Table S2)

INTERVIEW GUIDE A: PATIENTS WITH CHRONIC DISEASES

INTRODUCTION (5 minutes)

Thank you for agreeing to participate in this study. Before we begin, I want to remind you about a few important things:

- This is a confidential conversation, and your responses will remain anonymous.
- There are no right or wrong answers; I'm interested in your honest experiences and perspectives.
- You can skip any question you're uncomfortable answering, and you can ask me to clarify anything.
- With your permission, I'll record this conversation to ensure accuracy. The recording will be stored securely and heard only by our research team.

Do you have any questions before we begin?

SECTION A: DEMOGRAPHICS AND GENERAL HEALTH (5 minutes)

The following demographic information should be collected using the separate demographic form. If not already completed, briefly obtain:

1. Age: ___
2. Gender: □ Male □ Female
3. Chronic disease diagnosis: _________
4. How long have you had this condition? _________
5. Current physical activity level: □ Low □ Moderate □ High

SECTION B: OVERALL EXERCISE EXPERIENCE (10 minutes)

Open-ended Primary Questions:

1. Overall Exercise Experience
    - "Can you describe your overall experience with physical activity and exercise? How would you characterize your exercise journey over time?"

    Probes if needed:

    - Has your exercise engagement changed over time?
    - What has been easy about exercising for you?
    - What has been difficult?
2. Current Exercise Patterns

- "Tell me about your current exercise routine. What activities do you engage in, and how often?"

Probes if needed:

- What types of exercise do you do?
- How frequently do you exercise?
- Has your routine changed recently?
- Are you satisfied with your current activity level?

SECTION C: NURSING SUPPORT AND INTERACTIONS (20 minutes)

3. Nursing Support Overview
    - "Can you describe your experience with nursing support related to physical activity and exercise? How have nurses supported—or not supported—your exercise engagement?"

    Probes if needed:

    - Which nurses have been involved in your care?
    - What is your relationship like with these nurses?
    - When have you discussed exercise with nurses?
4. Nursing Education and Guidance
    - "What kinds of information or guidance have nurses provided you about exercise? How have those conversations been?"

    Probes if needed:

    - What did they explain to you about exercise?
    - How did you feel about the information provided?
    - Was it helpful? Why or why not?
    - Did the nurses explain the benefits of exercise for your specific condition?
5. Personalized Exercise Planning
    - "Have nurses helped you develop or plan an exercise program specifically tailored to your situation? Tell me about that experience."

    Probes if needed:

    - How were goals set?
    - Did the nurses ask about your preferences, limitations, or concerns?
    - How realistic were the recommendations for your life?
    - Did you participate in planning, or were you just given recommendations?
6. Motivation and Encouragement

- "How has nursing support affected your motivation to exercise? Can you give me examples?"

Probes if needed:

- How do nurses encourage you?
- What kinds of feedback or recognition have you received?
- Has this affected your motivation?
- What would enhance your motivation?

7. Monitoring and Follow-Up
   - "How often do nurses follow up with you about your exercise? What do those follow-up interactions look like?"

Probes if needed:

- Do they ask about your exercise at appointments?
- How often do you discuss exercise with nurses?
- Is this enough for you?
- What would ideal follow-up look like?

SECTION D: BARRIERS AND FACILITATORS (15 minutes)

8. Barriers to Exercise Adherence
   - "What makes it difficult or challenging for you to exercise regularly? What are the main barriers you face?"

Probes if needed:

- Physical barriers: pain, fatigue, fear of worsening symptoms?
- Psychological: motivation, mood, confidence?
- Environmental: facilities, safety, time, weather?
- Social: family support, competing responsibilities?

9. How Nursing Has Addressed Barriers
   - "Have you discussed these barriers with your nurses? How have they helped you address them?"

Probes if needed:

- Did they offer solutions?
- Did those solutions work?
- What would have been more helpful?
- What barriers persist despite nursing support?

10. Facilitators to Exercise Adherence

- "What helps you maintain your exercise routine? What factors make it easier to exercise?"

Probes if needed:

- Personal factors: determination, perceived benefits, improved energy?
- Social factors: family support, exercise partners, group programs?
- Environmental: nearby facilities, accessible options, financial feasibility?
- Healthcare: nursing support, medical provider encouragement?

11. Role of Family and Social Support
    - "How has your family or others in your life supported—or hindered—your exercise efforts? Have nurses involved your family in supporting your exercise?"

Probes if needed:

- Does anyone exercise with you?
- Do they encourage you?
- Has nursing spoken to your family about their role in supporting exercise?
- How could family support be enhanced?

SECTION E: PERCEIVED HEALTH BENEFITS AND OUTCOMES (5 minutes)

12. Observed Health Benefits
    - "Have you noticed any health improvements or benefits from exercising? Tell me about those changes."

Probes if needed:

- Physical improvements: blood pressure, glucose levels, weight, strength, endurance?
- Emotional improvements: mood, anxiety, self-confidence?
- Functional improvements: ability to do activities you care about?
- Life satisfaction and quality of life?

13. Connection Between Exercise and Health
    - "How have these benefits—or lack of benefits—affected your commitment to exercise?"

Probes if needed:

- Do you see clear connections between your exercise efforts and health improvements?
- Are these improvements motivating?
- Have nurses helped you recognize or appreciate these benefits?

SECTION F: SATISFACTION AND RECOMMENDATIONS (10 minutes)

14. Appropriateness of Exercise Recommendations
    - "Do you feel that the exercise recommendations you received were appropriate for your abilities and situation?"

    Probes if needed:

    - Were they realistic given your health condition?
    - Did they account for your physical limitations?
    - Were they achievable within your life circumstances?
    - Were they too challenging or not challenging enough?
15. Desired Changes in Nursing Support
    - "If you could change anything about the nursing support you receive for exercise, what would it be? What would help you adhere better?"

    Probes if needed:

    - Different types of support?
    - More frequent contact?
    - Different communication methods?
    - More resources?
    - Different approach or style?
    - Involvement of other healthcare providers?
16. Impact of Exercise on Daily Life
    - "How has exercise affected your daily life and quality of life? What changes have you noticed?"

    Probes if needed:

    - Functional changes: energy, stamina, ability to do preferred activities?
    - Emotional changes: confidence, mood, sense of control?
    - Social changes: engagement with others, independence?
    - Overall satisfaction with life?
17. Understanding and Personalization
    - "Do you feel that your nurses understand your specific needs and circumstances related to exercise? How could that understanding be improved?"

    Probes if needed:

    - Do they ask about your life, preferences, and concerns?
    - Do recommendations feel personalized?
    - What would help them understand you better?
18. Most Valued Aspects of Nursing Support

- "What has been most helpful or meaningful about the nursing support you've received regarding exercise? What has made the biggest difference for you?"

Probes if needed:

- Specific strategies?
- Personal qualities of nurses?
- Style of communication?
- Consistency and availability?

SECTION G: FUTURE PERSPECTIVES (5 minutes)

19. Future Expectations and Recommendations
    - "What are your hopes and expectations for nursing support related to exercise in the future? What recommendations would you make to improve nursing support for exercise in chronic disease management?"

    Probes if needed:

    - Structured follow-up?
    - Better resources?
    - Involvement of other healthcare team members?
    - Access to exercise programs or facilities?
    - Different approach to motivation?
    - Education for nurses about exercise?
20. Advice to Others
    - "Would you recommend that other patients with chronic diseases commit to regular exercise? Why or why not? What would you tell them?"

    Probes if needed:

    - Benefits you've experienced?
    - Challenges you've overcome?
    - Importance of nursing support?
    - How to start?

Thank you very much for sharing your experiences with me. Your insights are valuable and will help us understand how nursing can better support exercise adherence in chronic disease management. Is there anything else you'd like to add that we haven't discussed?

INTERVIEW GUIDE B: NURSING PROFESSIONALS

INTRODUCTION (5 minutes)

Thank you for agreeing to participate in this study. Before we begin, I want to remind you about a few important things:

- This is a confidential conversation, and your responses will remain anonymous.
- There are no right or wrong answers; I'm interested in your honest perspectives and experiences.
- You can skip any question you're uncomfortable answering, and you can ask me to clarify anything.
- With your permission, I'll record this conversation to ensure accuracy. The recording will be stored securely and heard only by our research team.
- Please know that your participation will not influence employment evaluations or professional standing.

Do you have any questions before we begin?

SECTION A: DEMOGRAPHICS AND PROFESSIONAL BACKGROUND (5 minutes)

The following demographic information should be collected using the separate demographic form. If not already completed, briefly obtain:

1. Age: ___
2. Gender: □ Male □ Female
3. Nursing credentials: □ Diploma □ Bachelor's □ Master's □ Doctorate
4. Years of experience in nursing: ___
5. Years of experience in chronic disease management: ___
6. Current position/clinical setting: _________
7. Recent training in health promotion or patient education: □ Yes □ No

SECTION B: ROLE AND RESPONSIBILITIES (10 minutes)

Open-ended Primary Questions:

1. Conceptualization of Nursing Role in Exercise Promotion
    - "How do you conceptualize your role as a nurse in promoting physical activity and exercise among patients with chronic diseases? What is your understanding of this responsibility?"

    Probes if needed:

    - Is exercise promotion a priority in your nursing care?

- How important is this compared to other aspects of care?
- What is your vision of ideal nursing support for exercise?

2. Current Practice Approach
   - "Can you describe your current approach to promoting exercise with your patients? What strategies and interventions do you typically use?"

   Probes if needed:

   - How do you approach conversations about exercise?
   - What information do you provide?
   - How often do you address this?
   - What strategies have you found effective?

SECTION C: PATIENT ASSESSMENT AND INDIVIDUALIZATION (10 minutes)

3. Assessing Patient Needs and Barriers
   - "How do you assess your patients' current activity levels, barriers to exercise, and exercise-related needs? Walk me through how you approach this assessment."

   Probes if needed:

   - What questions do you ask?
   - How do you identify barriers?
   - How do you learn about individual preferences and concerns?
   - How do you tailor your approach based on this information?
4. Tailoring Interventions
   - "How do you tailor your exercise promotion strategies to individual patients? How do you account for different needs, limitations, and preferences?"

   Probes if needed:

   - Different recommendations for different patients?
   - How do you decide what approach will work for a particular person?
   - How do you involve patients in planning?
   - How do you adjust your approach if initial strategies aren't working?
5. Overcoming Common Barriers
   - "What are the most common barriers to exercise adherence that your patients face? How do you help them address these barriers?"

   Probes if needed:

   - Physical barriers: pain, fatigue, fear?
   - Psychological barriers: motivation, confidence, mood?
   - Environmental barriers: access, time, family support?
   - How effectively are you able to address these?
   - What barriers are most challenging?

SECTION D: EDUCATION, MOTIVATION, AND SUPPORT (10 minutes)

6. Health Education Approach
    - "How do you educate patients about the importance and benefits of exercise for their specific conditions? What is your educational approach?"

    Probes if needed:

    - What information do you provide?
    - How do you explain the connection between exercise and health outcomes?
    - How do you correct misconceptions or fears?
    - Do you use written materials, videos, demonstrations?
    - How effective is your educational approach?
7. Building Self-Efficacy and Confidence
    - "How do you help patients build confidence in their ability to exercise despite their chronic disease limitations? What strategies do you use?"

    Probes if needed:

    - Starting with small goals?
    - Positive feedback and reinforcement?
    - Graduated progression?
    - Addressing fears?
    - Celebrating successes?
8. Goal-Setting and Planning
    - "How do you work with patients to set exercise goals and develop exercise plans? Describe your approach."

    Probes if needed:

    - Collaborative vs. directive?
    - SMART goals?
    - Breaking into steps?
    - How realistic are the goals?
    - How often are goals reviewed and adjusted?
9. Motivation and Encouragement
    - "How do you motivate patients and sustain their commitment to exercise over time? What strategies are most effective?"

    Probes if needed:

    - Type of encouragement you provide?
    - How often do you encourage patients?
    - What works best for maintaining motivation?
    - How do you handle periods when motivation flags?
    - Use of peer support, group activities, or external incentives?

10. Monitoring and Follow-Up
    - "How do you monitor your patients' exercise adherence over time? What is your follow-up process?"

    Probes if needed:

    - Frequency of follow-up?
    - What do you assess during follow-up?
    - How do you respond if adherence is declining?
    - How do you adjust plans based on progress?
    - What are the barriers to consistent follow-up?

SECTION E: INTERDISCIPLINARY COLLABORATION (5 minutes)

11. Collaboration with Other Healthcare Providers
    - "How do you collaborate with other members of the healthcare team—physicians, physiotherapists, nutritionists, mental health providers—regarding exercise promotion and patient support?"

    Probes if needed:

    - Frequency and nature of collaboration?
    - Sharing of information about patient exercise progress?
    - Coordination of recommendations?
    - Interdisciplinary goal-setting?
    - What barriers exist to collaboration?
12. Family Involvement
    - "How do you involve family members in supporting patients' exercise engagement? What strategies do you use?"

    Probes if needed:

    - Educating family about exercise benefits?
    - Enlisting family as exercise partners or supporters?
    - Addressing family barriers to support?
    - How effective is family involvement?

SECTION F: CHALLENGES AND FACILITATORS (10 minutes)

13. Perceived Challenges and Facilitators to Effective Exercise Promotion
    - "What are the main challenges or obstacles you face in promoting exercise with your patients? What makes this work difficult?"

Probes if needed:

- Patient-related factors: resistance, low motivation, fears, competing demands?
- System factors: time constraints, workload, lack of resources, lack of organizational support?
- Knowledge or skill gaps on your part?
- Lack of access to exercise programs or facilities?

14. Perceived Effectiveness of Current Approach
    - "How effective do you perceive your current exercise promotion efforts to be? What percentage of your patients maintain regular exercise?"

Probes if needed:

- Satisfaction with outcomes?
- Variation in effectiveness across different patients?
- Factors associated with success vs. non-adherence?
- What would improve outcomes?

SECTION G: PROFESSIONAL DEVELOPMENT AND RESOURCES (8 minutes)

15. Training and Education Needs
    - "What professional development, training, or education would help you be more effective in promoting exercise? What knowledge or skills would be beneficial?"

Probes if needed:

- Exercise physiology?
- Behavior change theories?
- Motivational interviewing?
- Specific strategies for your patient population?
- Technology or tools?

16. Resource and Support Needs
    - "What resources, support, or changes would help you implement exercise promotion more effectively in your practice?"

Probes if needed:

- More time for patient education?
- Educational materials or tools?
- Access to exercise programs or facilities?
- Technology support?
- Organizational policies supporting exercise promotion?
- Interdisciplinary collaboration structures?
- Evaluation and feedback about effectiveness?

SECTION H: RECOMMENDATIONS AND FUTURE VISION (7 minutes)

17. Recommendations for Improving Exercise Promotion
    - "What recommendations would you make for improving nursing support for exercise adherence in chronic disease management? What would make the biggest difference?"

    Probes if needed:

    - Changes in nursing practice?
    - Organizational or systemic changes?
    - Education and training?
    - Resources or tools?
    - Collaboration and coordination?
    - Patient education materials or programs?
18. Vision for Ideal Practice
    - "If you could design the ideal approach to nursing support for exercise in chronic disease management, what would it look like? What would you do differently?"

    Probes if needed:

    - Structure and frequency of interactions?
    - Content and approach?
    - Resources available?
    - Team composition and collaboration?
    - Outcome measures?
19. Perceived Impact of Exercise on Patient Outcomes
    - "What impact do you observe when patients successfully adhere to exercise recommendations? How do you see this affecting their health and quality of life?"

    Probes if needed:

    - Physical health improvements?
    - Psychological benefits?
    - Functional improvements?
    - Quality of life changes?
    - Patient satisfaction?

Thank you very much for sharing your perspectives and experiences. Your insights are valuable and will help us understand how to better support nursing efforts in promoting exercise adherence. Is there anything else you'd like to add that we haven't discussed?
